# A novel algorithm for 3-D visualization of electrogram duration for substrate-mapping in patients with ischemic heart disease and ventricular tachycardia

**DOI:** 10.1371/journal.pone.0254683

**Published:** 2021-07-14

**Authors:** Mustafa Masjedi, Christiane Jungen, Pawel Kuklik, Fares-Alexander Alken, Ann-Kathrin Kahle, Niklas Klatt, Katharina Scherschel, Jürgen Lorenz, Christian Meyer

**Affiliations:** 1 Department of Cardiology, Angiology and Intensive Care, EVK Düsseldorf, cNEP, cardiac Neuro- and Electrophysiology Research Consortium, Düsseldorf, Germany; 2 Institute of Neural and Sensory Physiology, cNEP, cardiac Neuro- and Electrophysiology Research Consortium, Heinrich Heine University Düsseldorf, Düsseldorf, Germany; 3 Department of Cardiology, Leiden University Medical Center, Leiden, Netherlands; 4 DZHK (German Centre for Cardiovascular Research), Partner Site Hamburg/Kiel/Lübeck, Germany; 5 Department of Cardiology, University Heart & Vascular Centre, University Hospital Hamburg-Eppendorf, Hamburg, Germany; 6 Department of Cardiology, Asklepios Hospital St. Georg, Hamburg, Germany; 7 Department of Cardiology, Schoen Hospital Neustadt, Neustadt in Holstein, Germany; 8 Faculty of Life Sciences, Department of Biomedical Engineering, Applied Science University Hamburg, Hamburg, Germany; University of Minnesota, UNITED STATES

## Abstract

**Background:**

Myocardial slow conduction is a cornerstone of ventricular tachycardia (VT). Prolonged electrogram (EGM) duration is a useful surrogate parameter and manual annotation of EGM characteristics are widely used during catheter-based ablation of the arrhythmogenic substrate. However, this remains time-consuming and prone to inter-operator variability. We aimed to develop an algorithm for 3-D visualization of EGM duration relative to the 17-segment American Heart Association model.

**Methods:**

To calculate and visualize EGM duration, in sinus rhythm acquired high-density maps of patients with ischemic cardiomyopathy undergoing substrate-based VT ablation using a 64-mini polar basket-catheter with low noise of 0.01 mV were analyzed. Using a custom developed algorithm based on standard deviation and threshold, the relationship between EGM duration, endocardial voltage and ablation areas was studied by creating 17-segment 3-D models and 2-D polar plots.

**Results:**

140,508 EGMs from 272 segments (n = 16 patients, 94% male, age: 66±2.4, ejection fraction: 31±2%) were studied and 3-D visualization of EGM duration was performed. Analysis of signal processing parameters revealed that a 40 ms sliding SD-window, 15% SD-threshold and >70 ms EGM duration cutoff was chosen based on diagnostic odds ratio of 12.77 to visualize rapidly prolonged EGM durations. EGMs > 70 ms matched to 99% of areas within dense scar (<0.2 mV), in 95% of zones within scar border zone (0.2–1.0 mV) and detected ablated areas having resulted in non-inducibility at the end of the procedure. Ablation targets were identified with a sensitivity of 65.6% and a specificity of 94.6% avoiding false positive labeling of prolonged EGMs in segments with healthy myocardium.

**Conclusion:**

The novel algorithm allows rapid visualization of prolonged EGM durations. This may facilitate more objective characterization of arrhythmogenic substrate in patients with ischemic cardiomyopathy.

## Introduction

Ventricular tachycardia (VT) in patients with ischemic cardiomyopathy (ICM) is a major cause of sudden cardiac death [1]. Implantable cardioverter defibrillators do not prevent arrhythmia occurrence and antiarrhythmic drug therapy is often limited or discontinued due to side effects. In consequence, catheter ablation is increasingly becoming an important therapeutic option for these patients, which can effectively reduce VT occurrence [2]. The arrhythmogenic substrate that is targeted during VT ablation is located predominantly at the border between normal and scarred myocardium [3]. This border is characterized by healthy tissue interspersed with fibrous scars. Electrophysiologically, this results in heterogeneous and slow conduction, thereby facilitating reentry mechanisms, the most common VT mechanism in ICM. Local electrograms (EGM) with abnormal characteristics serve as a surrogate parameter for slow conducting zones during substrate-based VT ablation and might improve the characterization of possible arrhythmogenic ablation targets [4,5]. Several EGM-based algorithms are well established in current mapping systems visualizing EGM amplitude and level of fractionation [6–8]. However, none of these algorithms currently display EGM duration, which can be an important surrogate of myocardial slow conduction [9]. Still, estimation of true EGM duration with a fixed set of parameters is challenging due to the great variability of EGM morphologies within regions of scar [7,8]. Moreover, as manual identification of EGM duration during sinus rhythm is time-consuming and prone to inter-operator variability, an automated algorithm may reduce procedure time. Whether and how this impacts procedural outcome is not known. However, in patients undergoing catheter ablation of scar-related VT prolonged procedure time has been linked with increased hospital mortality [10]. Therefore, in this study, we developed a novel algorithm for 3-dimensional (3-D) visualization of EGM duration relative to the 17-segment American Heart Association model [11] to objectively identify potential arrhythmogenic areas of slow conduction in patients with ICM and VT in an automated and time-saving way.

## Materials and methods

### Study enrollment

Sixteen patients with ICM undergoing high density mapping (HDM) guided VT ablation were retrospectively analyzed. Only patients with complete endocardial voltage maps during sinus rhythm were included. As a retrospective analysis of clinically acquired data was performed anonymously, the Institutional Review Board waived the need for patient written informed consent. The study was approved by the ethics committee of the Ärztekammer Hamburg, Germany, and conforms to all principles outlined by the Declaration of Helsinki. Antiarrhythmic medication was continued prior to ablation at the operator’s discretion. Patients were followed-up in our outpatient clinic 3 months after ablation and subsequently every 3 to 6 months.

### Electrophysiological study and ablation

Detailed procedural methods for VT ablation in patients with ICM have been described before [2]. Briefly, the catheter setting consisted of a 6F quadripolar diagnostic catheter, which was placed in the right ventricular apex to perform programmed ventricular stimulation using a standard protocol. Furthermore, one decapolar catheter (Inquiry, St. Jude Medical, Saint Paul, MN, USA) was placed in the coronary sinus and served as a reference for the three-dimensional HDM which was performed using the Rhythmia mapping system (Boston Scientific, Marlborough, MA, USA) during sinus rhythm. For this, an expandable 64-polar mini basket catheter (Orion, Boston Scientific) comprising of eight splines with eight electrodes (electrode spacing 2.5 mm, electrode surface area 0.4 mm^2^) was maneuvered into the left ventricle via an atrial transseptal puncture, using a fixed curve long sheath (SL0, 8-F; St. Jude Medical, USA; for the ablation catheter) and a long steerable sheath (Agilis large curl, 8.5-F, St. Jude Medical, for the mini basket catheter) [2]. Electrogram annotation was conducted automatically by the mapping system using the following criteria for beat acceptance: (1) cycle length stability: ±10 ms; (2) stability of relative timing of reference electrograms: ±5 ms; (3) respiration gating: automatic measurement and filtering of motion above maximum inspiration movement by change of impedance of the ECG-electrodes; (4) electrode location stability: 2–3 mm [12]. The maximum projection distance of the electrode to the captured anatomic geometry was set to 3 mm [13]. Substrate maps were evaluated as complete when the entire chamber anatomy was recreated with best achievable electrode-tissue contact and scar borders were defined clearly. Complete chamber mapping with the basket-catheter was subsequently verified with the single-tip ablation catheter. Additional activation mapping was performed whenever VT was inducible and hemodynamically tolerated. If patients were hemodynamically instable (defined as a mean arterial blood pressure <50 mmHg) and the VT was terminated, pace mapping during sinus rhythm was performed. The induced VT was defined as the clinical VT when cycle length (cycle length within 20 ms) and morphology matched previous recordings (12-lead-electrocardiogram and/or device recordings). Heparin was administered to maintain an activated clotting time of > 300 sec during the whole procedure.

Ablation (maximum power of 50 W at an irrigation rate of 17–30 ml/min) was performed using a 3.5-mm externally open irrigated-tip ablation catheter (IntellaNav MIFI, Boston Scientific) to target areas in order to achieve VT non-inducibility. Radiofrequency current were conducted in a temperature-controlled mode with a maximum temperature of 48°C and a maximum output of 20–50 W depending on the location and expected myocardial thickness [14]. Programmed stimulation was performed from the right ventricular apex adopting basic drive cycle lengths of 510, 440, and 370 ms with up to three extrastimuli. For inducible and mappable VT, ablation in the critical isthmus of the VT was performed. Critical isthmus sites were defined as the part of the VT circuit which is delimited by conduction barriers showing the smallest activation region. Critical sites of the circuit served as ablation target [14]. For inducible and not-inducible but hemodynamically instable VT, regions with late potentials and local abnormal ventricular activity (LAVA) were targeted [14,15]. The procedural endpoint was defined as noninducibility and elimination of late potentials as well as LAVA and fractionated potentials [14]. All ablation sites leading to VT termination were annotated for subsequent analysis during algorithm development.

### Analysis of electroanatomic substrate maps

Electroanatomical mapping datasets were exported from the Rhythmia system as MAT-file and were imported into a custom-developed software, which was implemented in a MATLAB (MathworksInc, Natick, MA) environment. 3-D high-density voltage maps were recreated and bipolar EGM were filtered using a bandpass filter (30–250 Hz) and a notch filter at 50 Hz for offline substrate analysis [16]. For reconstruction of electroanatomic 3-D maps a fill threshold of 5 mm as default setting of the Rhythmia system was set [17]. All mapping data were acquired during stable sinus rhythm, data from pace mapping were not used for substrate analysis. Voltage cutoffs for healthy myocardium > 1.0 mV, scar border zone 0.2–1.0 mV and dense scar < 0.2 mV were set [2]. All electroanatomic 3-D maps were transformed according to the 17-segment model to divide the left ventricle for EGM analysis [11]. The landmarks for this segmentation were set in cooperation with 2 experienced electrophysiologists. For substrate analysis a mean voltage value of each segment was calculated and compared to EGM duration as well as segments in which ablation was performed.

### Development and validation of the algorithm for estimation of electrogram duration

Since the bipolar EGM characteristics are influenced in timing and morphology by the size of the scar, border zone as well as location of the scar [18] an algorithm based on standard deviation (SD) and threshold were developed to automatically detect onset and offset of various single waveforms [19]. First, a sliding SD-window in milliseconds (test range: 10–45 ms) was defined that can indicate changes in the amplitude and frequency of EGM. Briefly, SD values were calculated within a moving window of predefined length across neighboring sample points for each individual local EGM to transform the signal into corresponding SD-curve. Second, a SD-threshold in percent (test range: 10–50%) was defined and applied on maximum value of the SD-curve. Onset and offset of EGM duration were identified by the interval of the SD-threshold value and SD-curve. Third, an EGM duration cutoff in milliseconds (test range: 60–80 ms) was applied as a threshold for automatized classification of abnormally prolonged EGM durations [20]. Based on the above-mentioned signal processing parameter tested, corresponding EGM duration maps were created and segmented according to the 17-segment model [11,21]. For each segment a mean EGM duration value (mean of all mapping points per segment) was calculated. In order to select best parameter set of signal processing (sliding SD-window/SD-threshold/EGM duration cutoff) predicting ablation site, correlation between mean EGM duration and segments with ablation as a known marker of arrhythmogenic substrate was conducted [22]. Diagnostic odds ratio (DOR) was used as a measure of test performance combining the strengths of sensitivity and specificity [23]. In this study, the DOR represents the odds that abnormal segments (> EGM duration cutoff) will be ablated compared to normal or healthy classified segments (no ablated areas). Thus, we used a parameter set that resulted in the highest DOR allowing the best possible sensitivity and/or specificity. Moreover, for each EGM duration map the percentage of prolonged EGM duration areas that matched to dense scar areas, border zone areas and ablated areas was determined.

### Statistical analysis

All continuous variables were tested for normal distribution using the Shapiro-Wilk test. Parametric data are expressed as mean ± standard error of the mean. Analysis was performed by chi-square test and binomial testing for comparison of proportions using probability parameters of 0.5 (2-sided) and 0.95 (1-sided). A P value < 0.05 was considered statistically significant. Logistic regression was performed to determine parameter sets marked by the highest DOR. All analyses were performed using SPSS (version: 25.0.0.0).

## Results

### Patient and procedural characteristics

Patient and procedural characteristics are summarized in Tables 1 and 2, respectively. Patients presented with a mean age of 66 ± 2.4 years, (94% male) and a mean left ventricular ejection fraction of 31 ± 2%. A prior VT ablation was performed in six patients (30%). A total of 23 VTs were detected in 16 procedures and consecutively analyzed (1.4 VTs ± 0.22 per patient). A mean of 7.931 ± 3.898 points per substrate map were taken during a mean mapping time of 57.01 ± 6.66 min. The mean VT cycle length was 375.38 ± 22.11 ms. A mean of 133 ± 87.21 ablation points were applied per patient. Scar was located in the following regions: 12.5% (2/16) at apex, 6.25% (1/16) at anterior septal, 18.75% (3/16) at inferior septal, 37.5% (6/16) at inferior basal, 12.5% (2/16) at inferior lateral and 12.5% (2/16) at anterior lateral. Procedural success with the endpoint of VT non-inducibility and elimination of late potentials as well as LAVA and fractionated potentials was achieved in all patients.

**Table 1:** Study population.

Characteristics	Values
**Patient characteristics**	**n = 16**
Sex, male, n (%)	15 (95)
Age at time of ablation (years)	66 ± 2.4
Left ventricular ejection fraction (%)	31 ± 2
ICD, n (%)	15 (95)
ICD for primary prevention	6 (35)
ICD for secondary prevention	9 (60)
**Comorbidities**	
Ischemic cardiomyopathy	16 (100)
Hypertension	10 (63)
Atrial fibrillation	12 (75)
Hyperlipidemia	13 (65)
Chronic kidney disease	9 (56)
Diabetes mellitus	1 (6)
Active smoking	5 (31)
**Medication, n (%)**	
Beta-receptor blocker	16 (100)
Amiodarone	5 (31.25)
Number of antiarrhythmic drugs: 1/2	10 (63)/5 (31)
ACEI/ARB	15 (94)
Aldosterone antagonist	6 (40)
Oral anticoagulation	14 (88)
**VT**	
History of VT (months)	35 ± 12
Patients with previous VT ablation, n (%)	6 (30)

ICD, Implantable cardioverter defibrillator; ACEI, Angiotensin converting enzyme inhibitor; ARB, Angiotensin receptor blocker; VT, Ventricular tachycardia. Values are mean ± s.e.m. or number (percent).

**Table 2:** Procedural data.

Procedural data	Values
VTs per patient, n	1.43 ± 0.22
Hemodynamically tolerated during the procedure, n (%)	2 (12.5)
Procedure duration (min)	222 ± 11
Fluoroscopy time (min)	17.26 ± 1.34
Dose area product (cGycm²)	1160 ± 205
RF application time (sec)	2565 ± 305
Ablation points per patient, n	133 ± 87.21
Substrate based ablation, n (%)	14 (87.5)
Points per substrate map, n	7.931 ± 3.898

RF, radiofrequency. Values are mean ± s.e.m. or number (percent).

### Analysis of voltage maps

In total, 272 segments relative to 17-segment American Heart Association model were extracted and analyzed from sixteen 3-D voltage maps of patients with ICM and VT. Using pre-defined voltage cutoffs, 65.4% (178/272) of segments were classified as healthy, 25% (68/272) as border zone and 9.6% (26/272) as dense scar. Ablation points were set in 111 segments, of which 39.6% (44/111) were located in healthy myocardium, 45.5% (51/111) in border zone areas and 14.4% (16/111) in dense scar.

**Fig 1:**
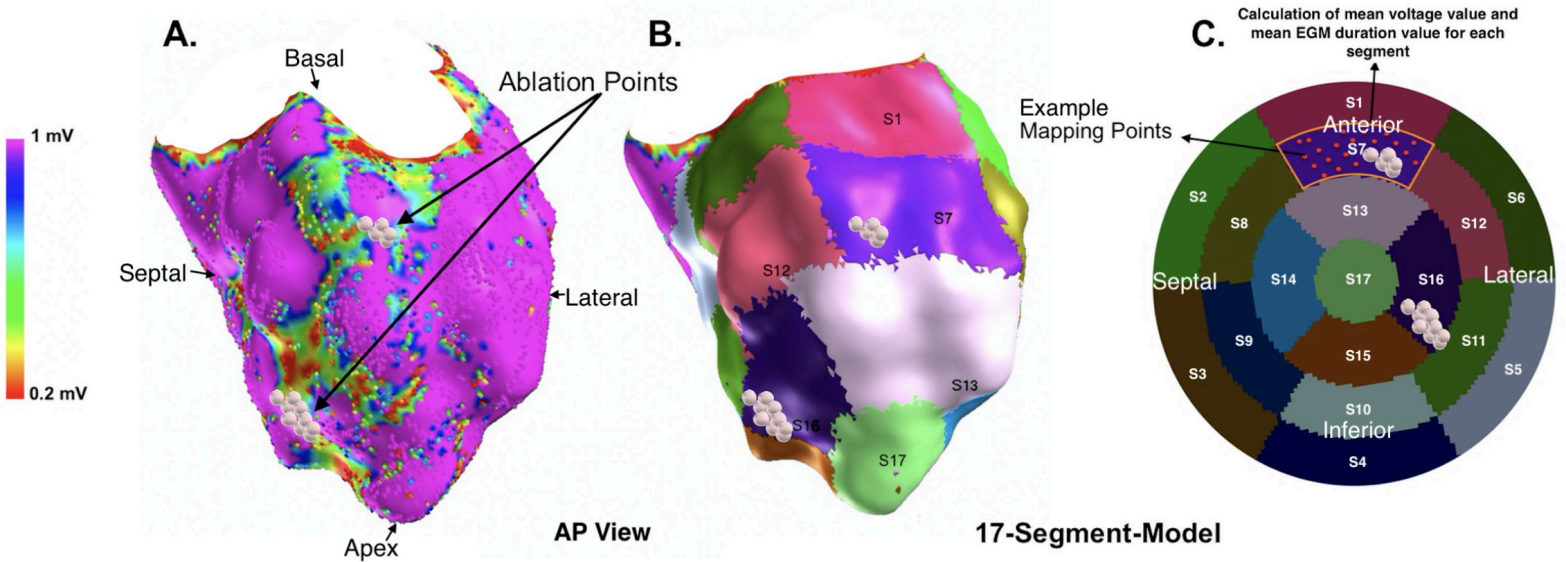
Illustration of the segmentation method for electroanatomical left ventricular voltage map analysis during sinus rhythm. (A) Color-coded areas of dense scar (< 0.2 mV, red), scar border zones (0.2–1.0 mV, yellow, green and blue) and healthy myocardium (> 1.0 mV, purple) are displayed in a patient with prior myocardial infarction and scar-related ventricular tachycardia undergoing catheter ablation. (B and C) The 17-segment-model was applied to the 3-dimensional map and 2- dimensional polar plot for further analysis of the localization of set ablation lesions (white points). The clinically documented VT was induced (cycle length 500 ms) and successfully terminated by ablation. The mean EGM duration and mean voltage value based on all EGM per segment (for example red points in segment 7) was calculated. Correlation between voltage, EGM duration and ablation were conducted segment by segment.

However, the 39.6% (44/111) of ablation points that were set in areas of healthy myocardium were located close to segments of border zone (binomial test, 2-sided, n = 111, P = 0.02). For voltage-based ablation target prediction a sensitivity of 50.4% and specificity of 68.34% were calculated retrospectively (chi-square value 3.606, p = 0.0576). Illustration of segmentation method for left ventricular voltage map analysis is in Fig 1 provided.

### Analysis of signal processing parameters

**Fig 2:**
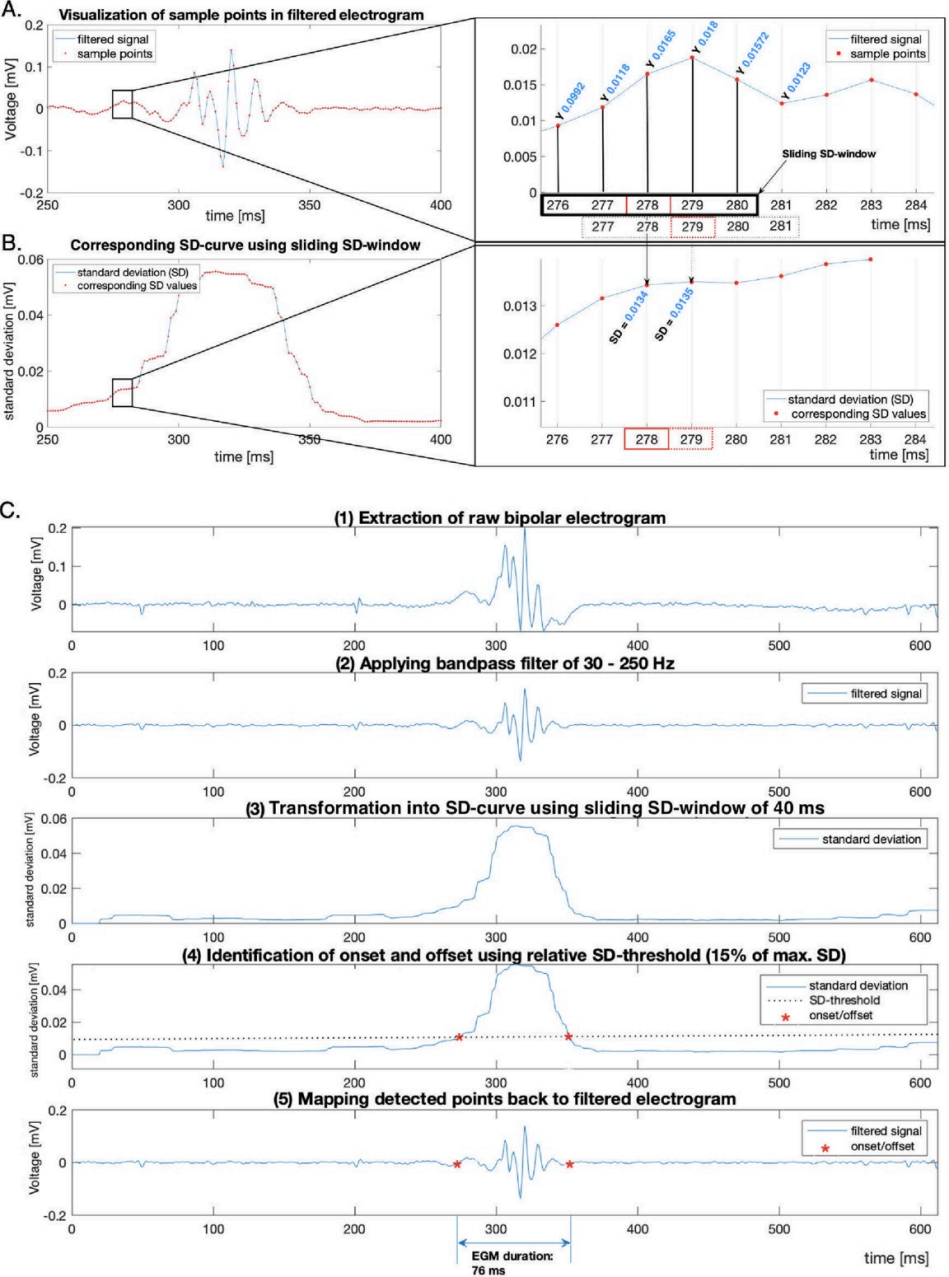
Method and schematic visualization of electrogram duration estimation using sliding SD-window and SD-threshold. (A) Visualization of filtered electrogram with its sample points (red dots) recorded with bipolar electrodes from a single beat. Sample points with corresponding local voltage are displayed at a rate of 1 millisecond (left). Magnification of the labelled interval (right). A sliding standard deviation (SD) window was applied to calculate SD over a moving window (black box) of predefined length across neighboring sample points. The dotted box indicates the method of consecutive SD value determination. (B) The corresponding SD values generate the SD-curve for further signal processing (left). Magnification of the labelled interval (right). The calculated SD value corresponds to a specific time unit (red box) in the moving window and is transformed into SD-curve. (C) Schematic visualization of workflow of signal processing parameter estimating EGM duration. After extraction of the raw bipolar electrogram during sinus rhythm (1), a bandpass filter of 30–250 Hz was applied (2). A sliding SD-window of 40 ms length calculates the SD across the electrogram (3). Onset and offset (red stars) were detected when 15% of the maximum SD (dotted line) was reached (4). Detected points (red stars) were transferred to the initially filtered electrogram to demonstrate onset and offset. The interval between onset and offset results in an EGM duration of 76 ms (5).

**Fig 3:**
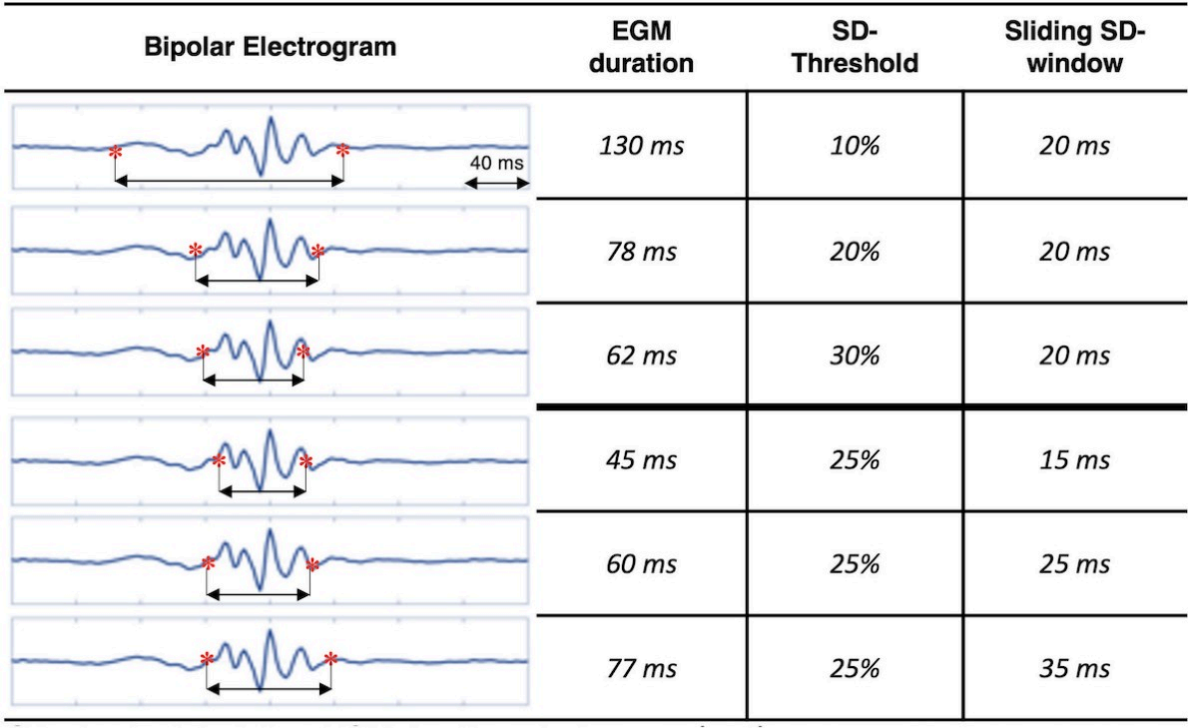
Visualization of Effects on electrogram duration estimation using various sliding SD-window and SD-threshold during sinus rhythm. Estimation of electrogram (EGM) duration decreases with rising SD-threshold: At SD-threshold = 10%, EGM duration is overestimated, whereas at SD-threshold = 30% EGM duration is underestimated by using constant SD-window size of 20 ms. Estimation of EGM duration increases with rising sliding SD-window: At sliding SD-window = 15 ms, EGM duration is underestimated, whereas at sliding SD-window = 35 ms EGM duration is overestimated by using constant SD-threshold of 25%.

**Fig 4:**
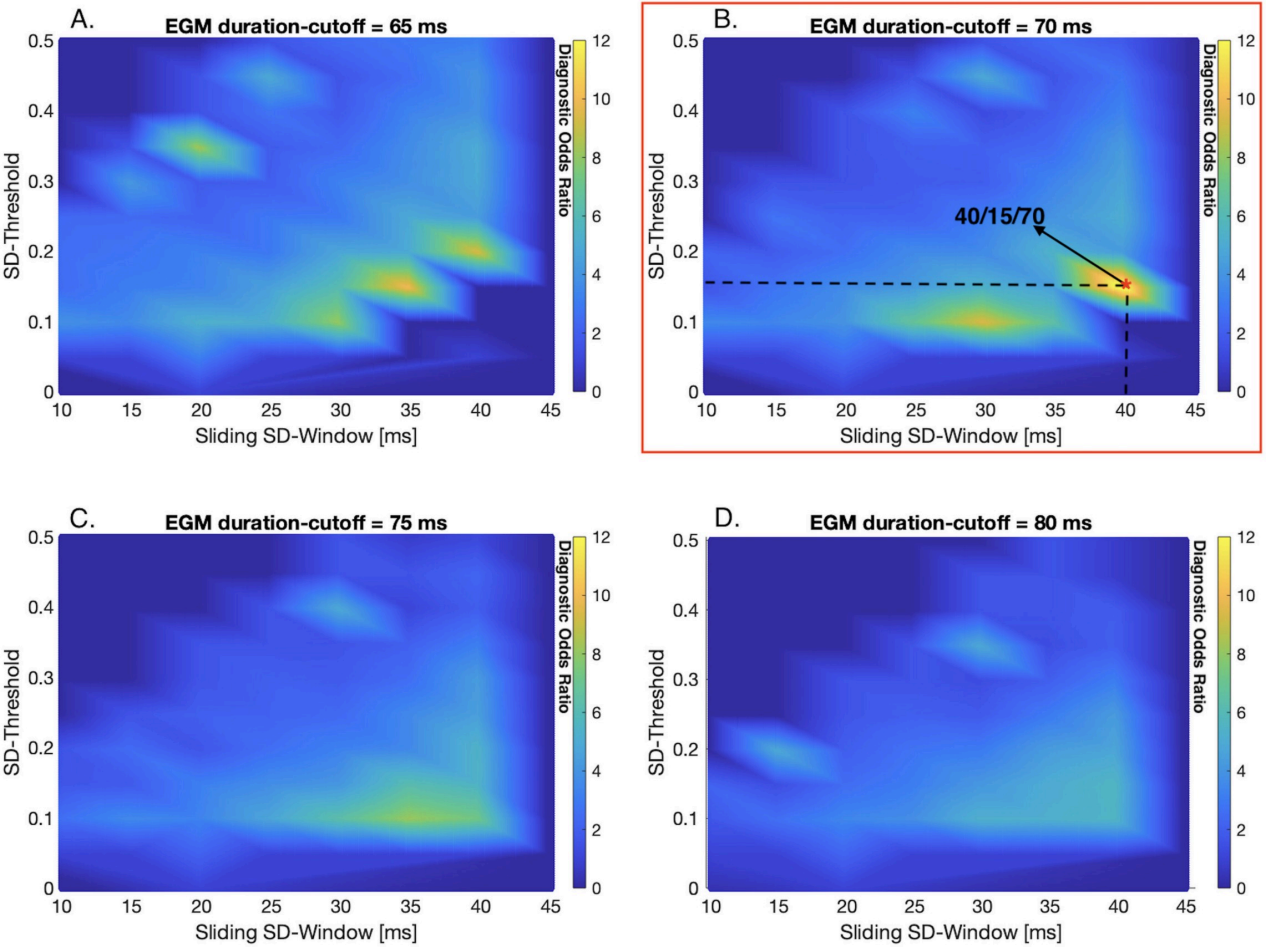
Diagnostic odds ratio color-coded maps demonstrate validation of signal processing parameters. According to the highest diagnostic odds ratio (DOR) value, the signal processing parameters sliding SD-window (test range: 10–45 ms) and SD-threshold (test range: 0.1–0.5) are validated. The color-coded scale represents linear interpolation of DOR, with yellow being the highest and blue the lowest. (A) The parameter sets 35/10/65 and 40/20/65 (SD-window size/SD-threshold/EGM duration cutoff) result in a DOR of 10.11. (B) The parameter set 40/15/70 results in the highest DOR of 12.77 (red asterisk). This parameter set leads to a DOR of 12.77, describing a 12.77-times higher probability that ablation points were set in areas with a mean EGM duration > 70 ms compared to areas with an EGM duration ≤ 70 ms (95% Cl: 3.0848–56.1523, P = 0.0005). (C) The parameter set 35/10/75 results in a DOR of 8.19. (D) The parameter set 40/10/80 results in a DOR of 5.53.

The estimation of EGM duration using signal processing parameter is demonstrated in Fig 2. The usage of different signal processing parameters has an effect on the EGM duration estimation. While high SD-thresholds are associated to underestimation of EGM duration, high sliding SD-window are related to overestimation (Fig 3). Detailed validation of signal processing parameters and their influence on the DOR are presented in Fig 4. The highest DOR between EGM duration map relative to 17-segment American Heart Association model and ablated segments was achieved using the following set of signal processing parameters: 40 ms SD-window size, 15% SD-threshold and 70 ms EGM duration cutoff (40 ms/15%/70 ms). Using these parameters results in a DOR of 12.77 (95% Cl: 3.0848–56.1523, P = 0.0005), which describes a 12-times higher probability for ablation points to be located in areas with a mean EGM duration > 70 ms compared to areas with an EGM duration ≤ 70 ms.

### Analysis of electrogram duration maps

**Fig 5:**
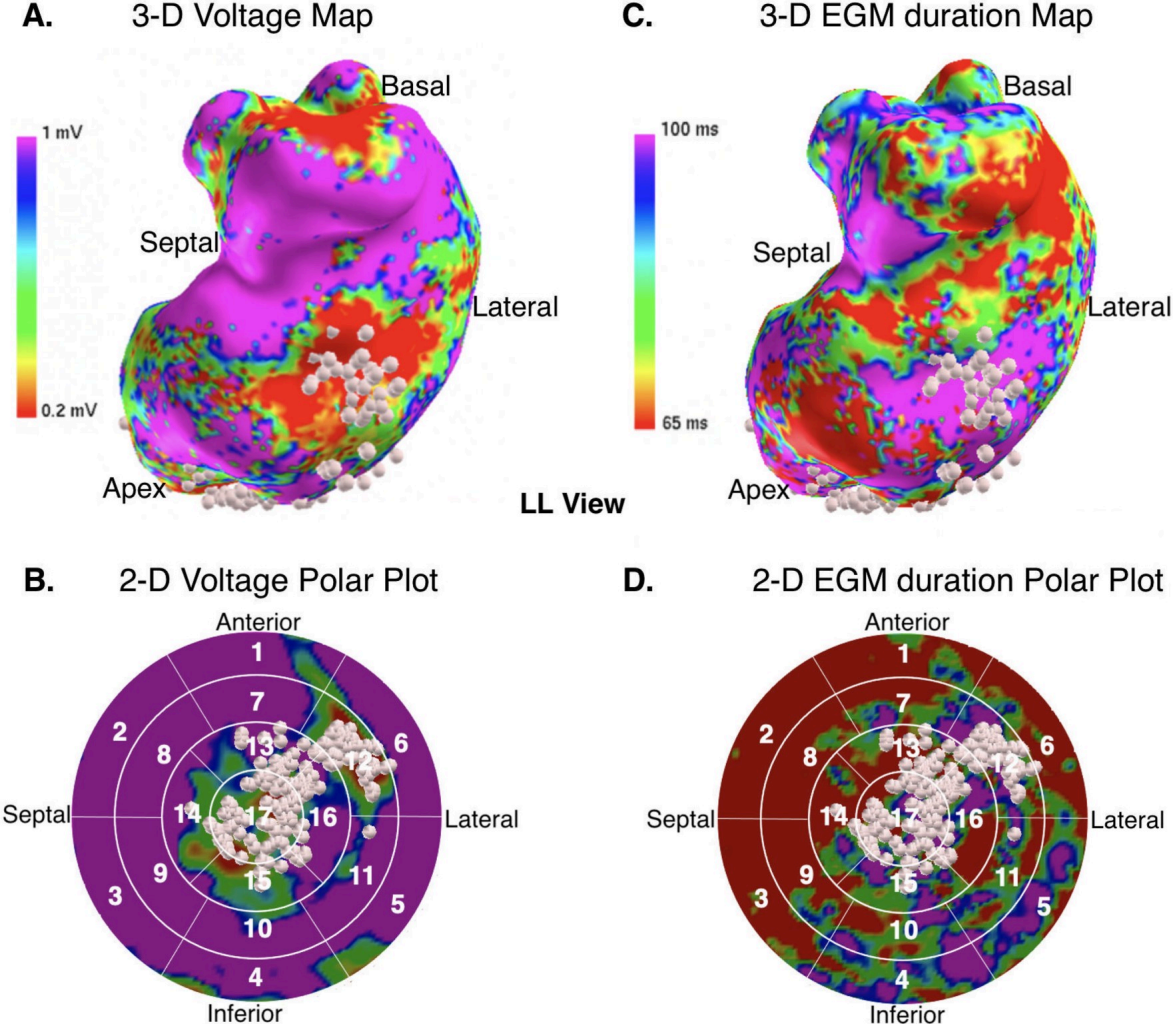
Image integration of a left ventricular 3-D-electroanatomical voltage and electrogram duration map into the 17-segment model. (A) Left ventricular 3-dimensional voltage map (left) and 3-dimensional electrogram (EGM) duration map (right) in a patient with scar-related ventricular tachycardia during sinus rhythm demonstrating that majority of ablation points (white points) were performed in areas with dense scar (red), scar border zone (yellow, green and blue) and long EGM duration (purple). The clinical documented VT was induced (cycle length: 320 ms) and terminated by ablation. (B) Electro-anatomical 2-dimensional polar plot translation of voltage map (left) and electrogram duration map (right) demonstrating the feasibility of automated EGM duration-based identification of low voltage (< 1 mV) areas and ablation targets in segments 12,13,16 and 17.

By using the designated parameter set (40 ms/15%/70 ms), the EGM duration algorithm classified prolonged EGM in 58.5% (160/272) of segments. In total, the mean EGM duration was >70 ms in 95% of areas in which ablation points were set. Ablation points were set more frequently in areas with a mean EGM duration > 70 ms compared to areas with EGM duration ≤ 70 ms (binomial test, 2-sided, n = 111, P = 0.001). This resulted in a 65.6% sensitivity and 94.6% specificity for ablation target prediction (chi-square value 26.098, p = 0.001). Analyzing prolonged EGM distribution in voltage maps, 99% of segments with dense scar had a mean EGM duration >70 ms (binomial test, 1-sided, n = 26, P = 0.264). In 95% of voltage border zones (0.2–1 mV) the mean EGM duration was > 70 ms (binomial test, 1-sided, n = 68, P = 0.124). In segments with healthy myocardium, 36.5% overlapped to prolonged EGM duration. The feasibility of the EGM duration map for automated visualization and characterization of border zone and dense scar area, in which ablation was performed, is illustrated in Fig 5.

### Follow-up

After a mean follow-up of 146 ± 24 days 15 of 16 patients (93.75%) had an arrhythmia free survival. One patient experienced a VT during follow up and underwent a further VT ablation procedure.

## Discussion

This study describes a novel algorithm allowing rapid 3-D visualization of EGM duration relative to the 17-segment American Heart Association model [11] for a more objective characterization of the arrhythmogenic substrate in patients with ICM. The resulting approach (1) automatically identifies and displays prolonged EGM durations on a 3-D high-density map and (2) allows precise analysis of the relationship between EGM duration and ablation targets.

### Automated determination of EGM duration

About a decade ago elimination of local abnormal ventricular activities has been found to be associated with superior survival free from recurrent VT [4]. Since then, several ablation approaches have been developed and substrate modification has become a valuable tool for state-of-the-art treatment in patients with scar-related VT [4,5]. However, VT ablation outcome still needs to be improved and these approaches are often prone to inter-operator variability. Objective identification of prolonged EGM duration as an important surrogate parameter for slow conduction might upgrade the armamentarium to improve procedural success and long-term patient outcome.

Critical isthmus sites were defined as the part of the VT circuit which is delimited by conduction barriers showing the smallest activation region [14]. In comparison to the isthmus site, the entrance and the exit site of the circuit as important pieces of the substrate puzzle are associated with slower conduction velocity and with prolonged LAVA [24,25]. Therefore, EGM duration has been used as a complementary surrogate parameter to characterize slow conduction zones [26]. Still, the great variability of abnormal EGM morphologies in scar-related areas makes establishment of a fixed set of signal processing parameters for an automated algorithm to estimate EGM duration challenging. Therefore, other algorithms have assessed the level of fractionation to identify the critical isthmus [7]. However, this results in an overestimation of EGM duration and thus, falsely reports slow conduction within healthy myocardium [7]. In our study, we have developed an algorithm that is based on SD and threshold. This has the advantage to be independent from EGM morphologies, which is influenced by the size as well as the location of the scar and the border zone [18]. The SD of an EGM indicates changes in the amplitude and frequency that is the purpose of a signal segmentation [19]. The fix threshold offers an automated way to objectively estimate onset and offset of EGM duration. Moreover, visualization of EGM duration relative to the 17-segment American Heart Association model [11] allows validation of the SD-based signal processing parameter in a more accurate manner and a novel way to analyze abnormal EGM duration on a 3-D map and in relation with other imagine modalities.

### EGM duration and slow conduction

Experimental and clinical studies demonstrated the relationship between EGM duration, slow conduction and critical VT isthmus sites [27,28]. Still, defining a cutoff for abnormal EGM duration is essential for the detection of slow conduction zones, which are potential ablation targets. Historically, EGM duration cutoffs were defined by manual measurement from the earliest onset of electrical activity to the decay artifact produced by the amplified filtered signal [20,29]. With this definition EGM duration cutoffs of > 50 ms in patients with VTs [29] or even > 70 ms in patients without obvious structural heart disease [20] were found to predict slow conduction areas. Newer publications have used algorithms to measure EGM duration. For example, Zeppenfeld et al. used a “peak to peak” algorithm that detects the distance between first sharp and last sharp peak. Based on 10 control patients with healthy left ventricles an EGM duration cutoff of > 40 ms was determined. By using these cutoffs [8,20,29], it was demonstrated that prolonged EGM are widely distributed at VT sites in general. In our approach, we determined an EGM duration cutoff of 70 ms to be the best parameter set with a DOR of 12.77. As 95% of patients with healthy left ventricle have an EGM duration of <70 ms [20], this indicates that our algorithm is specific for abnormal EGM in the here presented patient population. If the EGM duration cutoff would be decreased to 40 ms, slow conduction zones would be over-estimated resulting in loss of specificity. If the EGM duration cutoff would be increased to 80 ms or higher, this would result in an under-estimated zone of slow conduction and decreased sensitivity.

### EGM duration and high-density mapping

The visualization of EGM duration as a 3-D map was demonstrated for the first time in a porcine model, which indicated a correlation with pathological VT substrates such as scar tissue [28]. Other studies have confirmed that most of the critical isthmus sites matched with areas of prolonged EGM duration and isolated potentials [8]. Noteworthy, these EGM duration maps were created with limited mapping points (< 200 mapping points per patients) using a 4-mm-tip mapping catheter with 2-mm ring-electrodes and 1-mm inter-electrode space. When data interpolation between the points is performed to improve the quality of display, unmapped areas are only estimates of far field EGM duration [30]. This problem can now be addressed by using high-density maps which allow to acquire easily > 2000 mapping points per patient. Our novel ultra-high-density EGM duration maps are based on more than 4000 mapping points (mean 7.931 ± 3.898) resulting in an accurate estimation and rapid 3-D visualization of prolonged EGM duration relative to the 17-segment American Heart Association [11] in order to identify slow conduction zones. In comparison to abnormal voltage data, EGM duration shows a higher sensitivity (abnormal voltage: 50.4% vs. EGM duration: 65.6%) and specificity (abnormal voltage: 68.34% vs. EGM duration: 94.6%) in ablation EGM duration may allow for an advanced analysis of the relationship between abnormal EGM characteristics, abated areas and procedural success.

### Limitations

There are several limitations to this study. First, this study represents a single-center experience only enrolling a limited number of patients in whom only a mean number of 1.4 VT was induced per patient. This might be related to current heart failure therapy and sedation during the procedure which needs to be considered in the interpretation of the data [31]. Nevertheless, the aim of the present study was to show the feasibility of the developed EGM duration algorithm. Second, a systematic detection of double potentials was beyond the scope of the developed algorithm. As long as the amplitude of the corresponding SD do not exceed the SD-threshold, double potentials were not considered in EGM duration estimation. Therefore, depending on signal amplitudes some clinically relevant double potentials might have been missed by the algorithm. Third, local EGM characteristics and voltages are dependent on direction and rate of ventricular activation [32]. The effects of wavefront activation were beyond the scope of the present study. Also, the presented algorithm did not incorporate the scar location relative to wavefront activation, which can influence bipolar EGM characteristics including EGM duration. The "one acquisition-two maps" technique [13] might be useful to address this question in future studies. Furthermore, although a reasonably high specificity was achieved, EGM duration still has only modest sensitivity in predicting the ablation targets. Therefore, incorporating the analysis of the direction and rate of ventricular activation might be useful to deepen the related mechanistic understanding and improve the sensitivity by using EGM duration analysis. Finally, only one mapping system was used and the used set of signal processing parameters may not be applicable to other mapping systems.

## Conclusion

The algorithm presented in this study allows a rapid 3-D visualization of prolonged EGM durations relative to the 17-segment American Heart Association model [11]. This facilitates objective characterization of the arrhythmogenic substrate and may improve substrate modification in patients with ICM and VT.

## Supporting information

S1 File EGM duration file of all patients relative to the 17-segment-model using different signal processing parameter. (ZIP)
